# Key performance indicators score (KPIs-score) based on clinical and laboratorial parameters can establish benchmarks for internal quality control in an ART program

**DOI:** 10.5935/1518-0557.20170016

**Published:** 2017

**Authors:** José G. Franco Jr, Claudia G. Petersen, Ana L. Mauri, Laura D. Vagnini, Adriana Renzi, Bruna Petersen, M.C. Mattila, Vanessa A. Comar, Juliana Ricci, Felipe Dieamant, João Batista A. Oliveira, Ricardo L.R. Baruffi

**Affiliations:** 1 Center for Human Reproduction Prof. Franco Jr., Ribeirao Preto, Brazil; 2 Paulista Center for Diagnosis, Research and Training, Ribeirao Preto, Brazil

**Keywords:** KPI, internal quality control, clinical pregnancy, IVF, ICSI, AMH

## Abstract

**Objective:**

KPIs have been employed for internal quality control (IQC) in ART. However, clinical KPIs (C-KPIs) such as age, AMH and number of oocytes collected are never added to laboratory KPIs (L-KPIs), such as fertilization rate and morphological quality of the embryos for analysis, even though the final endpoint is the evaluation of clinical pregnancy rates. This paper analyzed if a KPIs-score strategy with clinical and laboratorial parameters could be used to establish benchmarks for IQC in ART cycles.

**Methods:**

In this prospective cohort study, 280 patients (36.4±4.3years) underwent ART. The total KPIs-score was obtained by the analysis of age, AMH (AMH Gen II ELISA/pre-mixing modified, Beckman Coulter Inc.), number of metaphase-II oocytes, fertilization rates and morphological quality of the embryonic lot.

**Results:**

The total KPIs-score (C-KPIs+L-KPIs) was correlated with the presence or absence of clinical pregnancy. The relationship between the C-KPIs and L-KPIs scores was analyzed to establish quality standards, to increase the performance of clinical and laboratorial processes in ART. The logistic regression model (LRM), with respect to pregnancy and total KPIs-score (280 patients/102 clinical pregnancies), yielded an odds ratio of 1.24 (95%CI = 1.16-1.32). There was also a significant difference (p<0.0001) with respect to the total KPIs-score mean value between the group of patients with clinical pregnancies (total KPIs-score=20.4±3.7) and the group without clinical pregnancies (total KPIs-score=15.9±5). Clinical pregnancy probabilities (CPP) can be obtained using the LRM (prediction key) with the total KPIs-score as a predictor variable. The mean C-KPIs and L-KPIs scores obtained in the pregnancy group were 11.9±2.9 and 8.5±1.7, respectively. Routinely, in all cases where the C-KPIs score was ≥9, after the procedure, the L-KPIs score obtained was ≤6, a revision of the laboratory procedure was performed to assess quality standards.

**Conclusion:**

This total KPIs-score could set up benchmarks for clinical pregnancy. Moreover, IQC can use C-KPIs and L-KPIs scores to detect problems in the clinical-laboratorial interface.

## INTRODUCTION

Systems to monitor clinical and laboratorial performance have gained much importance in medical practice (^Leandro *et al.*, 2005^; ^Vermeulen *et al.*, 2008^; ^Salinas *et al.*, 2010^). The assessment of clinical or surgical practices should take advantage of techniques developed for controlling processes in the manufacturing industry.

A performance indicator or key performance indicator (KPI) is a type of performance measurement. Any process, whether in a biomedical or non-biomedical field, can be subject to inherent deviations from the optimum or from established limits. These deviations may lead to defective end-products or, in the medical field, defective patient care. Monitoring, which is a process able to identify deviations, and then being able to act should such deviation exceed certain limits, plays an important role in avoiding adverse consequences and maintaining optimal performance.

It should be noted that published studies (PubMed research) about KPIs and ART are practically nonexistent. Information is usually obtained through classes on laboratorial quality control, or opinions of authors expressed in chapters of books. In this way, KPIs have been employed for internal quality control (IQC) in IVF/ICSI programs, using indicators such as oocyte fertilization, cleavage embryo rates, percentage of top-quality embryos, etc. KPIs analyses can be plotted and compared with established limits for the mean and standard deviation values, so that deviations can be easily recognized as warnings or action points, but possibility a false interpretation of a KPI drop could not be excluded. Laboratory conditions (temperature, pH, humidity, air quality, culture media, equipment, etc.) could modify these variables and negatively impact the outcomes. Since the KPIs data is available after some weeks, the impact of a problem not detected in the laboratory may potentially affect outcome for a significant period.

However, in no time, clinical KPIs (C-KPIs) such as age, AMH and number of oocytes collected are added to laboratory KPIs (L-KPIs) for analysis, even though the final endpoint is the evaluation of clinical pregnancy rates.

The purpose of this study was to develop a total KPIs-score (C-KPIs+L-KPIs) with the power to identify individual benchmarks, as well as to analyze the laboratory performance during different situations. The KPIs scores strategy application could result in an immediate evaluation of the patient's clinical and laboratory performance in the ART cycle. In addition, internal quality control benchmarks could be evaluated.

## MATERIALS AND METHODS

### Patients' characteristics and inclusion criteria

In this prospective cohort study, 280 patients (36.4±4.3years) were submitted to ICSI cycles during 2015-2016. All patients met the following criteria: body mass index (BMI) between 20-30kg/m^2^, regular menstrual cycles, both ovaries present, no history of ovarian surgery, no severe endometriosis and no evidence of endocrine disorders. The study was authorized by the local ethical committee, and a written informed consent was obtained from all recruited subjects.

**Figure 1:**
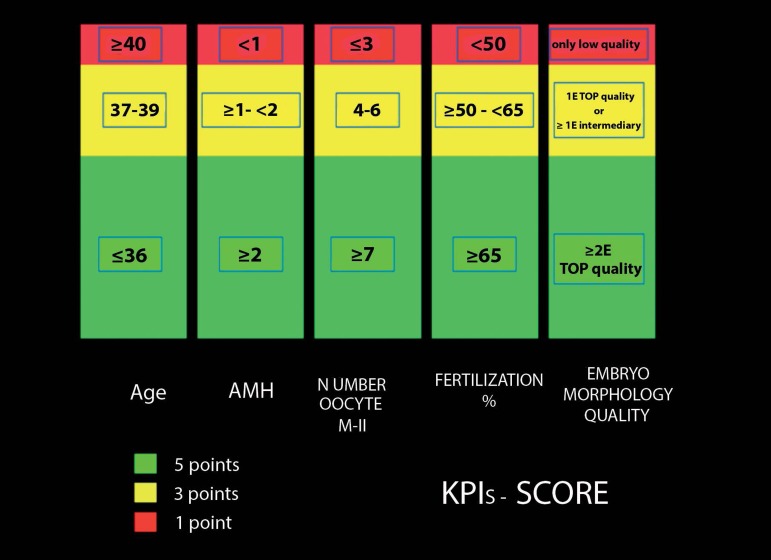
The KPIs- score system.

The KPIs score was obtained by analysis of age, AMH (ng/ml), number of metaphase-II oocytes, fertilization rates and morphological quality of embryonic lot (MQEL). The maximum total KPIs score was 25 points (age ≤36, AMH ≥2, number of oocytes on metaphase II ≥7, fertilization rate ≥65%, number of top quality embryos ≥2), the minimum total KPIs score was 5 points (age ≥ 40, AMH <1, number of oocytes on metaphase II ≤3, fertilization rate <50%, only low quality embryos (see for more details: Figure 1).

### AMH measurement

A venous blood sample for an AMH measurement was taken before the scheduled treatment (minimum of 30 days), during the early follicular menstrual cycle phase in all the women. AMH was measured using an enzymatically amplified 2-site immunoassay kit (AMH Gen II ELISA/modified, Beckman Coulter Inc.). These AMH levels were obtained after modifying the methodology suggested by ^Han *et al.* (2014)^. The lowest detection limit of this assay is 0.01ng/ml, whereas the maximum intra- and inter-assay variation coefficients were 3.3% and 6.5%, respectively. To minimise the likelihood of bias in the assay, all sera were processed in duplicate during the same day, using the same measurement kits, and it was done by the same operator. Low- and high-level controls were included in each assay.

### Ovarian stimulation protocol

The patients were subjected to 2 schemes of controlled ovarian stimulation:

- GnRH-agonist protocol: the pituitary downregulation began during the luteal phase of the previous menstrual cycle with GnRH-a leuprolide acetate (leuprolide acetate; Lupron^®^; Abbott, Brazil) at a dose of 1mg/day for 14 days. The ovaries were then stimulated with a fixed dose of 75-375IU of recombinant FSH (r-FSH; Gonal F^®^; Serono, Brazil) and 75IU/day of recombinant *luteinising hormone* (r-LH; Luveris^®^; Serono, Brazil) for a period of 7 days. On day 8 of the ovarian stimulation, follicular development was monitored by a transvaginal ultrasound at 7MHz. The r-FSH dose was modified according to the ovarian response, and r-LH supplementation was increased to 150 IU/day when one or more follicles measuring ≥10mm in diameter were found.
- GnRH-antagonist protocol: On day 3 of the cycle, ovarian stimulation was induced with a fixed dose of 75-375IU of r-FSH and 75IU/day of r-LH for a period of 5 days. On day 8 of the menstrual cycle (day 6 of ovarian stimulation), the follicular development was monitored by transvaginal ultrasound at 7MHz. The r-FSH dose was modified according to the ovarian response, and the r-LH supplementation was increased to 150IU/day when 1 or more follicles measuring ≥10mm in diameter were found. The GnRH-ant (cetrorelix; Cetrotide^®^; Serono, Brazil) was started at a dose of 0.25mg/day s.c., when at least 1 follicle of ≥14mm was seen in the ultrasound scan.

To induce final oocyte maturation in both protocols (GnRH-a and GnRH-ant), 250µg of recombinant human chorionic gonadotropin (r-hCG; Ovidrel; Serono, Brazil) was administered s.c., when at least 2 follicles reached a mean diameter of ≥17mm. Except at the risk of developing SHO when GnRH-a was added at 1500IU doses of hCG for final oocyte maturation. GnRH-a and GnRH-ant were administered until the day of the r-hCG injection. Oocyte retrieval was performed by transvaginal aspiration under ultrasound guidance 34-36 hours following the r-hCG injection.

### Metaphase II oocytes

The retrieved oocytes were incubated in culture medium for 1-2hour(s). Cumulus cells were removed by exposing the oocytes to hyaluronidase, after which coronal cells were removed and the denuded oocytes were classified according to their level of maturation. Oocytes with the first polar body, i.e., in metaphase II (MII) were considered to be mature and the total number used in ICSI was used as a benchmark for the KPIs-score.

### Fertilization rates

Fertilization was seen 16-19h after the procedure, to determine the presence or absence of pronuclei. A normal fertilization process was defined on the basis of the formation of two distinct pronuclei. The percentage of fertilized oocytes was used as benchmark for the KPIs-score.

### Morphological quality of the embryonic lot

The morphological quality of the embryonic lot was evaluated routinely from 48 to 72 after injection at the cleavage stage, depending on the day of transfer, day 2 or day 3, respectively.

**Embryos transferred at day 2: They were scored according to the following criteria:**
**-Grade 6 (top quality):** embryos presenting 4 cells (blastomeres), with symmetrical blastomeres, with no blastomere fragmentation and with one nucleus in each blastomere;
**-Grade 5 (top quality):** embryos presenting 4 cells (blastomeres), with symmetrical blastomeres, with no blastomere fragmentation and absence of one nucleus in each blastomere;
**-Grade 4 (intermediary quality):** embryos without 4 cells (blastomeres), with symmetrical blastomeres and with no blastomere fragmentation;
**-Grade 3:** embryos presenting 4 cells (blastomeres), with irregular blastomeres and with ≥10 <25% of blastomere fragmentations;
**-Grade 2:** embryos presenting 4 cells (blastomeres), with irregular blastomeres, with ≥25% of blastomere fragmentations;
**-Grade 1:** embryos without 4 cells (blastomeres), with irregular blastomeres, with ≥10 <25% of blastomere fragmentations;
**-Grade 0:** embryos without 4 cells (blastomeres), with irregular blastomeres, with ≥25% of blastomere fragmentations.

**Embryo transferred on day 3: They were scored per the following criteria:**
**-Grade 5 (top quality):** embryos presenting 8 cells (blastomeres), with symmetrical blastomeres and with no blastomere fragmentation.
**-Grade 4 (intermediary quality):** embryos without 8 cells (blastomeres), with symmetrical blastomeres and with no blastomere fragmentation.
**-Grade 3:** embryos presenting 8 cells (blastomeres), with irregular symmetrical blastomeres and with ≥10 < 25% blastomere fragmentation.
**-Grade 2:** embryos presenting 8 cells (blastomeres), with irregular symmetrical blastomeres and with ≥25% blastomere fragmentation.
**-Grade 1:** embryos without 8 cells (blastomeres), with irregular symmetrical blastomeres and with ≥10 <25% blastomere fragmentation.
**-Grade 0:** embryos without 8 cells (blastomeres), with irregular symmetrical blastomeres and ≥25% blastomere fragmentation.

**KPI points evaluation:**
**-5 points=** the embryonic lot presented ≥2 embryos scored Grade 6 or Grade 5;
**-3 points=** the embryonic lot presented 1 embryo scored Grade 6 or Grade 5, or 2 embryos scores Grade 4;
**-1 point=** the embryonic lot presented only embryos Grade 3,2,1,0 or 1 embryo Grade 4.
**Clinical pregnancy:** Clinical pregnancy was defined as the presence of a gestational sac in the uterine cavity with a heartbeat at 6 gestation's week, detected by ultrasonography.
**Statistical analysis:** The data was analyzed using the Stats Direct statistical software (Cheshire, UK). For dichotomous variables, correlations were performed using the logistic regression model (LRM).

### RESULTS

#### Clinical pregnancy benchmark

The total KPIs-score (C-KPIs+L-KPIs) was correlated with the presence or absence of clinical pregnancy. The relationship between the C-KPIs score and L-KPIs score was analyzed to establish quality standards to increase the performance of the clinical and laboratory work in ART.

The logistic regression model with respect to clinical pregnancy and total KPIs-score (280 patients/102 clinical pregnancy) yielded an odds ratio of 1.24 (95%CI = 1.16-1.32). Also, there was a significant difference (p<0.0001) with respect to the total KPIs mean score among the group of patients with clinical pregnancy (total KPIs-score=20.4±3.7) and the group without clinical pregnancy (total KPIs-score=15.9±5). Clinical pregnancy probabilities (CPP) can be obtained using the LRM (prediction key) with the total KPIs-score as a predictor variable; therefore, total KPIs-score 25/CPP=70% (95%CI = 59%-79%); total KPIs-score=20/CPP=45% (95%CI = 38%-51%), total KPIs-score=15/CPP=22% (95%CI=16%-30%), total KPIs-score =10/CPP=9% (95%CI = 5%-15%), etc. (Table 1).

**Table 1:** Total KPIs-score and clinical probabilities

TOTAL KPIs-SCORE	CLINICAL PREGNANCY PROBABILITIES	CONFIDENCE INTERVAL
25	70%	59%-79%
24	65%	56%-74%
23	60%	51%-69%
22	55%	47%-63%
21	50%	42%-57%
20	45%	38%-51%
19	40%	33%-46%
18	35%	28%-41%
17	30%	24%-36%
16	26%	20%-32%
15	22%	16%-30%
14	18%	13%-26%
13	15%	10%-22%
12	13%	8%-20%
11	10%	6%-18%
10	9%	5%-15%
9	7%	4%-14%
8	6%	3%-12%
7	5%	2%-10%
6	4%	1.6%-9%
5	3%	1.2%-8%

#### C-KPIs and L-KPIs benchmarks

On the other hand, the mean C-KPIs score and L-KPIs scores obtained in the pregnancy group were 11.9±2.9 and 8.5±1.7, respectively. Routinely, in all cases in which the C-KPIs score was ≥9 after the procedure but the L-KPIs score obtained was ≤6 (approximately one standard deviation and half less than the mean value), a revision of the laboratory procedure was performed to check the quality standards.

## DISCUSSION

The KPIs system was used to detect early warning signals in gamete/embryo cultures. However, the final endpoint is the evaluation of clinical pregnancy rates, although the C-KPIs are not usually included in the final evaluation.

In this study, the C-KPIs was a product of the analysis of three variables that have a significant correlation with pregnancy rates (age, AMH and number of oocytes collected).

**C-KPIs Predictor variable/Age:** age is the most traditional factor negatively correlated with pregnancy and live birth rates (LBR) after IVF/ICSI. This association between female age and LBR was nonlinear, with marked decreases in LBR after 28, 35 and 38 years of age (^Vaegter *et al.*, 2017^). Some studies dichotomized age into two categories, <35 or ≥35 years (^Sharma *et al.*, 2002^). Women aged 35 years or older had significantly lower pregnancy rates when compared with women who were younger than 35 years. Others studies categorized the patients into four groups, i.e., 30, 30-34, 35-38 and 39-45 years (^Sabatini *et al.*, 2008^). Women among the 30 and 30-34 years age-categories had 3.2 and 2.8 higher pregnancy likelihood when compared with women in the age category of 39-45 years. The third study showed that women aged 30 years or older had lower pregnancy rates when compared with women in the 25-29 group (^Wang *et al.*, 2008^). The biological explanation for this decline in conceiving likelihood with increasing female age most likely lies in the diminished ovarian reserve, a decrease in both quantity and quality of oocytes, which is clinically relevant in women from their mid-30s (^Broekmans *et al.*, 2007^). Despite difficulties in establishing the ideal and precise intervals of age as a benchmark in the prediction of clinical pregnancy, this study used three categories (≤36, 37-39, ≥40 years), almost like those described by ^Sabatini *et al.* (2008)^.

**C-KPIs Predictor variable/AMH:** Attempts have also been made to correlate serum AMH levels with the occurrence of pregnancy in ART cycles. Values higher than 2.7ng/mL were associated with higher rates of implantation and pregnancy (^Silberstein *et al.*, 2006^). Also, the level of AMH is associated with LBR after IVF/ICSI in women with (extremely) low ovarian reserve. The LBR in women with AMH ≥0.4ng/ml was significantly higher than in women with AMH ≤0.4ng/ml (^Reijnders *et al.*, 2016^). AMH could serve as a tool in the pre-treatment counseling for pregnancy likelihood in women with (extremely) low ovarian reserve. ^Gleicher *et al.* (2016)^ described some relationship between levels of AMH and LBR: best LBR values (43-47 %) were obtained at AMH 3.5-7.0ng/ml; intermediate rates (32-41 %) at AMH of 1.5-3.0 and 7.5-9.0ng/ml, but even poor prognosis with AMH of ≤1.0ng/ml was still associated with 25-29 % live births. The low (<1), intermediate (≥1- <2) and high (≥2) AMH levels were defined as ranges for this C-KPIs score benchmark.

**C-KPIs Predictor variable/Number of oocytes retrieved:** Six studies reported on the association between the number of oocytes retrieved and pregnancy rates (^Strandell *et al.*, 2000^; ^Hart *et al.*, 2001^; ^Hunault *et al.*, 2002^; ^Sharma *et al.*, 2002^; ^Ottosen *et al.*, 2007^). Two studies categorized the data. One study dichotomized the number of oocytes into ≤5 and >5 oocytes retrieved (^Sharma *et al.*, 2002^). The other study used three categories: 1-5 oocytes, 6-10 and 11 or more oocytes (^Ottosen *et al.*, 2007^). Both studies found that women with more oocytes had higher likelihoods of pregnancy. ^Van Loendersloot *et al.* (2010)^ found a positive association between increasing number of oocytes retrieved and pregnancy likelihood after IVF, with an OR of 1.04 (95%CI = 1.02-1.07). The number of oocytes in metaphase II (≥7) was used as the best benchmark value, corresponding to values equal to or above the mean of those previously found in the patients who achieved clinical pregnancies in our clinic.

On the other hand, L-KPIs scores were associated with two variables (fertilization rate and the morphological quality of embryos):

**L-KPIs Predictor variable/Fertilization rate:** The fertilization rate of the oocyte is a KPI traditionally used in monthly, semi-annual and even annual IQC evaluations in ART. However, there are no studies defining abnormal situations and what measures were adopted to solve the problem. On the other hand, one, two, or three standard deviations from an average of ideal values are used to define this benchmark. However, this KPI is not directly linked to the prediction of clinical pregnancy and, consequently, of live births in ART; but it provides basic information on laboratory quality control. The percentage of fertilization (≥65%) used as best benchmark, corresponded to values equal to or above the mean of those previously observed in the patients who achieved clinical pregnancies in our clinic.

**L-KPIs Predictor variable/Morphological quality of embryonic lot:** Three studies evaluated the association between embryo quality and pregnancy rates after IVF (^Strandell *et al.*, 2000^; ^Hunault *et al.*, 2002^; ^Ottosen *et al.*, 2007^). One study classified embryo quality using two separate factors, evaluating the best and the second-best embryos in terms of stage of development and morphology score (^Hunault *et al.*, 2002^). The stage of development was described using three categories: Delayed, Appropriate and Advanced. The Advanced Stage was used as the reference category. Women in whom either the best or second-best embryo had a delayed or appropriate development had lower pregnancy likelihood when compared with women in whom either the best or second-best embryo was at an Advanced Stage of development. Lower morphology scores were also associated with lower pregnancy likelihoods. The second study reported that women with embryos at a higher developmental stage and morphology scores, combined into one predictor, had higher pregnancy likelihoods when compared with women at lower developmental stage and morphology score (^Ottosen *et al.*, 2007^). The third study used three other predictors for embryo quality: number of good quality embryos available, number of good quality embryos transferred and number of embryos suited for freezing (^Strandell *et al.*, 2000^). All three predictors were associated with higher pregnancy likelihoods after IVF. In all the studies, better embryo quality was associated with higher likelihoods of pregnancy, but since these studies used different factors or combinations of embryo factors to report embryo quality, it was not possible to pool the data and calculate a summary OR (^Vaegter *et al.*, 2017^). The number of embryos top ≥2 used as best benchmark corresponded to values equal to or higher than the mean of those found previously in the patients who achieved clinical pregnancies in our clinic.

**Application of the KPIs-score strategy:** The data from this study showed that the values of the total KPIs-score have an excellent correlation with the rates of clinical gestation, thus becoming an excellent predictor of the cycle analyzed. In this way, it is possible to establish an individualized prognosis for each patient, avoiding a prediction based on global gestational data obtained in the clinic. On the other hand, if there is no gestation, all efforts should be directed to an increase of the total KPIs-score in a new attempt. From the clinical point of view, the main suggestions should be directed to the models of ovarian stimulation, since the age and the AMH values are not susceptible to modifications. However, the isolated or comparative observation of C-KPIs and L-KPIs scores also provides important information for planning an upcoming ART cycle. When C-KPIs and L-KPIs scores are maximum but gestations do not occur, other causal factors could be investigated, such as difficulties in embryo transfer processes, presence of factors that would compromise endometrial receptivity or suspected problems in luteal phase supplementation. In the situation where the C-KPIs score is ≥9 and the L-KPIs score is ≤6 points, an immediate laboratory evaluation should be performed for the identification or not, of future laboratory problems. At that time, an analysis of the quality of gametes is advised, as well as a detailed discussion about possible changes introduced in the laboratory (new culture media, changes in the incubator system, culture plates, embryo transfer catheters, etc.). On the other hand, there are cases where the C-KPIs score was low and the L-KPIs score reached 10 points, the term employed is *positive inversion*, in which case the embryologist's job should be highlighted. However, concerns about embryologists' performance can be raised when an embryologist does not exceed the L-KPIs benchmark score 6 (when the C-KPIs score benchmark is always ≥9). This alarm signal can be used as an important instrument in the laboratory IQC. On the other hand, one of the limitations of the application of the KPIs-score strategy is that each clinic should establish its own key performance indicators and benchmarks.

## CONCLUSION

This total KPIs-score could set up benchmarks for clinical pregnancy. Moreover, IQC can use C-KPIs and L-KPIs scores to detect problems in the clinical-laboratorial interface.

## References

[r1] Broekmans FJ, Knauff EA, te Velde ER, Macklon NS, Fauser BC (2007). Female reproductive ageing: current knowledge and future
trends. Trends Endocrinol Metab.

[r2] Gleicher N, Kushnir VA, Sen A, Darmon SK, Weghofer A, Wu YG, Wang Q, Zhang L, Albertini DF, Barad DH (2016). Definition by FSH, AMH and embryo numbers of good, intermediate-
and poor-prognosis patients suggests previously unknown IVF
outcome-determining factor associated with AMH. J Transl Med.

[r3] Han X, McShane M, Sahertian R, White C, Ledger W (2014). Premixing serum samples with assay buffer is a prerequisite for
reproducible anti-Mullerian hormone measurement using the Beckman Coulter
Gen II assay. Hum Reprod.

[r4] Hart R, Khalaf Y, Yeong CT, Seed P, Taylor A, Braude P (2001). A prospective controlled study of the effect of intramural
uterine fibroids on the outcome of assisted conception. Hum Reprod.

[r5] Hunault CC, Eijkemans MJ, Pieters MH, te Velde ER, Habbema JD, Fauser BC, Macklon NS (2002). A prediction model for selecting patients undergoing in vitro
fertilization for elective single embryo transfer. Fertil Steril.

[r6] Leandro G, Rolando N, Gallus G, Rolles K, Burroughs AK (2005). Monitoring surgical and medical outcomes: the Bernoulli
cumulative SUM chart. A novel application to assess clinical
interventions. Postgrad Med J.

[r7] Ottosen LD, Kesmodel U, Hindkjaer J, Ingerslev HJ (2007). Pregnancy prediction models and eSET criteria for IVF patients-do
we need more information?. J Assist Reprod Genet.

[r8] Reijnders IF, Nelen WL, IntHout J, van Herwaarden AE, Braat DD, Fleischer K (2016). The value of Anti-Müllerian hormone in low and extremely
low ovarian reserve in relation to live birth after in vitro
fertilization. Eur J Obstet Gynecol Reprod Biol.

[r9] Sabatini L, Zosmer A, Hennessy EM, Tozer A, Al-Shawaf T (2008). Relevance of basal serum FSH to IVF outcome varies with patient
age. Reprod Biomed Online.

[r10] Salinas M, López-Garrigós M, Gutiérrez M, Lugo J, Sirvent JV, Uris J (2010). Achieving continuous improvement in laboratory organization
through performance measurements: a seven-year experience. Clin Chem Lab Med..

[r11] Sharma V, Allgar V, Rajkhowa M (2002). Factors influencing the cumulative conception rate and
discontinuation of in vitro fertilization treatment for
infertility. Fertil Steril.

[r12] Silberstein T, MacLaughlin DT, Shai I, Trimarchi JR, Lambert-Messerlian G, Seifer DB, Keefe DL, Blazar AS (2006). Mullerian inhibiting substance levels at the time of HCG
administration in IVF cycles predict both ovarian reserve and embryo
morphology. Hum Reprod.

[r13] Strandell A, Bergh C, Lundin K (2000). Selection of patients suitable for one-embryo transfer may reduce
the rate of multiple births by half without impairment of overall birth
rates. Hum Reprod..

[r14] Vaegter KK, Lakic TG, Olovsson M, Berglund L, Brodin T, Holte J (2017). Which factors are most predictive for live birth after in vitro
fertilization and intracytoplasmic sperm injection (IVF/ICSI) treatments?
Analysis of 100 prospectively recorded variables in 8,400 IVF/ICSI
singleembryo transfers. Fertil Steril.

[r15] van Loendersloot LL, van Wely M, Limpens J, Bossuyt PM, Repping S, van der Veen F (2010). Predictive factors in in vitro fertilization (IVF): a systematic
review and meta-analysis. Hum Reprod Update.

[r16] Vermeulen RP, Jessurun GA, Peels HO, Jaarsma T, Zijlstra F (2008). Clinical performance indicators for percutaneous coronary
intervention. Crit Pathw Cardiol.

[r17] Wang YA, Healy D, Black D, Sullivan EA (2008). Age-specific success rate for women undertaking their first
assisted reproduction technology treatment using their own oocytes in
Australia, 2002-2005. Hum Reprod.

